# The Greatest Hardship of the Assistant Medical Officer

**Published:** 1918-08-03

**Authors:** 


### PROMOTION IN THE ASYLUM SERVICE.

### The Greatest Hardship of the Assistant Medical Officer.

The Cambridge Asylum at Fulbourne has long been notorious for the little discretion that it allowed to its medical superintendent, who was scarcely allowed to have a broken pane of glass repaired without an express order from the Committee of Visitors. Dr. Rogers has now resigned his control, if control it could be called, of the institution, and after nearly thirty-five years' service has taken his pension and retired into private life. This happened some years ago, on the coming into operation of the Asylum Officers Superannuation Act. Dr. Rogers then retired, and his senior assistant, Dr. Thompson, was appointed in his stead. The affair was normal and proper; it excited no feeling and called for no remark. But after the lapse of years, things have come out that throw a lurid light upon the transaction. It seems that Dr. Rogers, who evidently has a keen commercial instinct, saw a way of making a little profit out of his retirement. He put before Dr. Thompson the alternatives that he (Dr. Rogers) might retire at once, or might postpone his retirement. If he retired at once, and Dr. Thompson were appointed in his stead, it would be so much to Dr. Thompson's advantage that he could afford to make Dr. Rogers a present of £300. In this view Dr. Thompson concurred. A formal agreement was drawn up by a solicitor instructed by Dr. Rogers, and was duly executed by the parties. Dr. Rogers thereupon resigned his post, and we may conjecture that he said and did nothing to prejudice Dr. Thompson's chance of succeeding him. Dr. Thompson did, in fact, succeed him, paid him the £300, and reigned in his stead; until one fine day the whole transaction came to light, and Dr. Thompson was invited to resign. This invitation, although it was repeated, he declined to accept, and the committee in its difficulty appealed to the Board of Control to know what should be done. Somewhat, it seems, to the surprise of the committee, the Board of Control replied that it took a very serious view of the matter, and regarded the conduct of the parties concerned as most reprehensible. Moreover, the Board of Control called the attention of the committee to the provisions of the Public Bodies Corrupt Practices Act, 1889, a pretty plain hint that, in the opinion of the Board, the provisions of this Act had been infringed. Upon the receipt of this opinion the committee plucked up its resolution and gave Dr. Thompson three months' notice to terminate his engagement.

The incident is an unsavoury one, and we should have passed it over in silence if it stood alone, but it does not. The whole system of the medical appointments to lunatic asylums is bad. We do not mean to say that such transactions as those between Dr. Rogers and Dr. Thompson are frequent: they are not; but the whole system is unsatisfactory.

The position of assistant medical officers is unsatisfactory. It is better than it was some years ago, but it is still very unsatisfactory. The position of assistant medical officers is not what it ought to be. It is far too subordinate. The medical superintendent, instead of being, as he should be, *primus inter pares*, has a despotic authority over his assistants, and disposes of them and of their treatment of the patients as he pleases. His assistants are not his colleagues, but his servants; and he is a very exceptional superintendent if he does not make them feel this. In some respects the assistant medical officer's position is inferior to that of the attendants, who are subordinates to him. They can live out. They can marry, have homes of their own, and live a normal family life. It is not long since no assistant medical officer in the country was in this position, and even now it is far too exceptional among them. The disparity of emolument between that of the superintendent and that of the assistant medical officer is absurdly exaggerated. This, again, is not as great as it was, but it is still far too great. It is frequent for the emolument of the superintendent to be six times that of his assistant, a disparity sometimes the reverse of their professional attainments, and out of proportion to their respective work and responsibility.

But the greatest hardship of the assistant medical officer, the reason the Asylum Service is generally shunned by able and ambitious young men, is the utter uncertainty of promotion. Promotion in the Asylum Service goes neither by seniority, nor by merit, nor by experience, nor by professional ability and eminence, but by haphazard. The brilliant young man, full of ardour and of energy, who has left his hospital and his university with a fine record of successes and of popularity, and who strives to carry the hospital tradition into the asylum and study his cases scientifically, finds himself frowned upon and cold-shouldered. He finds that scientific work is not wanted. He finds that the only way to be comfortable is to efface himself as much as possible and merely to say ditto to his superior officer, who is considered, and considers himself, so vastly superior. The natural consequence is that young men of ability and ambition shun the Asylum Service; or if they do take posts therein, they make haste to get out of a position so repellent. The further consequence is that asylums, being staffed by medical men who have no aspirations towards scientific work, who are without ambition, and whose interest in the medical side of their work, if it ever existed, is soon starved out, are scientifically stagnant. Alienism is the most difficult branch of medical science, it is true, but the backward state of the science is not due to its difficulty alone. The absence of any scientific work in asylums is due mainly to the absence of scientific spirit and scientific aspirations in the *personnel*. The little work that has been done by such men as Dr. Orr and Dr. Rows is solely microscopical, and the experience of the last fifty years has shown plainly

**Promotion in the Asylum Service—(*continued*).**

enough that the microscope is not the instrument by which a knowledge of insanity is to be attained.

Another urgent reason for the unpopularity of asylum posts among ambitious and enterprising young men is the utter uncertainty of promotion, and the total indifference displayed by the Committees of Visitors, who make the appointments, to the medical qualifications of the candidates and to their record of asylum experience. A man may spend fifteen or twenty of the best years of his life in the subordinate and humble position of assistant medical officer, a position in which he ranks below the steward and the clerk, and find himself at the end of that time no nearer the goal of his ambition, no nearer to a position in which he can marry and take upon himself the full duties and privileges of manhood and citizenship. Nay, he finds that the very length of his service tells against him. Committees look askance upon him, and reckon the number of years before he will take his pension. They assume that there must be some sufficient reason why he has not been successful before, and they pass him over in favour of some younger man. He subsides into the position of a "chronic" A.M.O., subordinate, it may be, to some man much younger and less experienced than himself. All the heart is taken out of him, and he becomes a mere mechanical hack, with no ambition but to get through his days with the least trouble to himself, and to enjoy the few pleasures that his solitary and isolated life permit of. There is no way out, for by long residence in an asylum he has lost the capability of adapting himself to general practice or for any other mode of life.

Nor do his grievances end here, for when a superior appointment does fall vacant, he has not only no assurance that it will be filled by the most competent, experienced, and able man, but he knows that it may be filled by the appointment of some outsider who has had only a few months or years of asylum experience, or, perhaps, has never seen the inside of an asylum in his life until he is appointed to take over its management. The influences by which the superintendency of an asylum is secured are various, but certainly a knowledge of insanity is the least of them. It is doubtful whether it is ever taken into consideration at all by the Committees of Visitors who make these appointments, and it is certain that the appointments are occasionally made on grounds that ought not to be considered at all. Vacancies in these superior positions occur but seldom. The positions are very important and very responsible, and it is the manifest duty of the Visitors to secure for them the best men that can be had; but an examination of the records shows that Committees of Visitors are not alive to their duties, and sometimes make appointments that ought not to be made. It is by no means unknown for a man to be appointed who has never had any experience of lunacy at all. In one case, the Medical Officer of Health for the borough was transferred to the superintendency of the borough asylum. In another, the clerk and steward, who had secured a paramount influence over the committee, secured the appointment of his nominee, a young man who could be trusted not to interfere with the dominance of the powerful official who secured his appointment. In more than one case a young man with a minimum of asylum experience has been appointed for no apparent reason except that he had married the daughter of a county magnate who had a powerful influence on the committee. Other cases could be cited, but these are enough to account for the notorious mediocrity of professional and general attainments that prevail among superintendents of asylums; and meanwhile the knowledge of insanity is at a standstill, or what advance is made in it is made by men outside of asylums. With the sole exception of the West Riding, *no addition to our knowledge of insanity has been made in the memory of any man now living by the superintendent of any public asylum in this country.*

There is only one effectual remedy for this discreditable state of things, and this remedy has been advocated before in the pages of THE HOSPITAL. It is that the Asylum Service should follow the Prison Service in being taken over by the State. It should become a State Service, and be administered by a department of State under the Ministry of Health, whose formation has been too long delayed. Promotion will then be regular, and according to merit, and when a young man on entering the Service is treated as an officer and a gentleman, and not as an upper servant; when he can look forward with confidence to promotion to higher and higher posts; when his future prospects are no longer a lottery with the great proportion of the chances against him; when he is not compelled to postpone his marriage indefinitely, or, perhaps, be prevented from ever marrying; then we may look to have a better class of men entering the Asylum Service and remaining in it; and then, perchance, the reproach of stagnation may at length be removed from English alienism.

